# Supplementary material for: The Repressor C Protein, Pf4r, Controls Superinfection of Pseudomonas aeruginosa PAO1 by the Pf4 Filamentous Phage and Regulates Host Gene Expression
Source: Viruses. 2021 Aug 15;13(8):1614. doi: 10.3390/v13081614 (PMC8402870; doi:10.3390/v13081614)
Supplement: Supplementary file 1 [file viruses-13-01614-s001.zip › repC_SupplemetaryFigures.pdf]

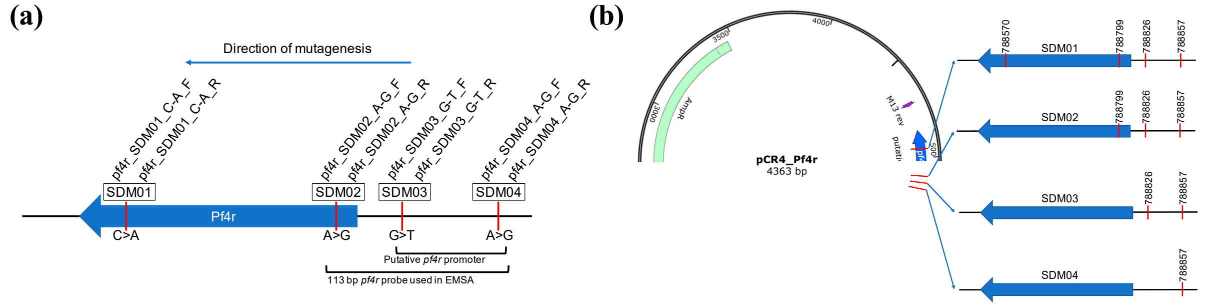

Figure S1. Sequence of site-directed mutagenesis and region of EMSA probe.
The direction of mutagenesis from right to left is indicated and performed sequentially using the respective primers (diagonal text) (a) to generate the construct of interest (b). Single nucleotide polymorphisms are indicated by the red vertical line and the nucleotide substitution. The *pf4r* was inserted into a pCR4 plasmid backbone containing an ampicillin resistance cassette as means for selection as depicted by the plasmid map.

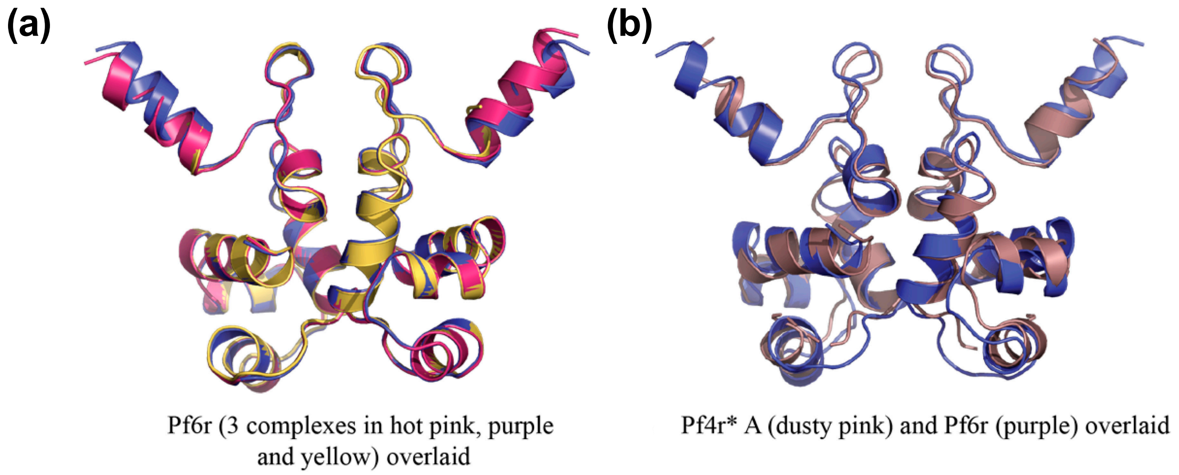

Figure S2. Crystal structure of Pf6r.
(a)Crystal structure of Pf6r showing an overlay of all 3 complexes in the unit cell overlaid (pink, yellow and blue), and an over-lay of Pf6r (Complex AB) with Type A complex for Pf4r* (dusty pink).

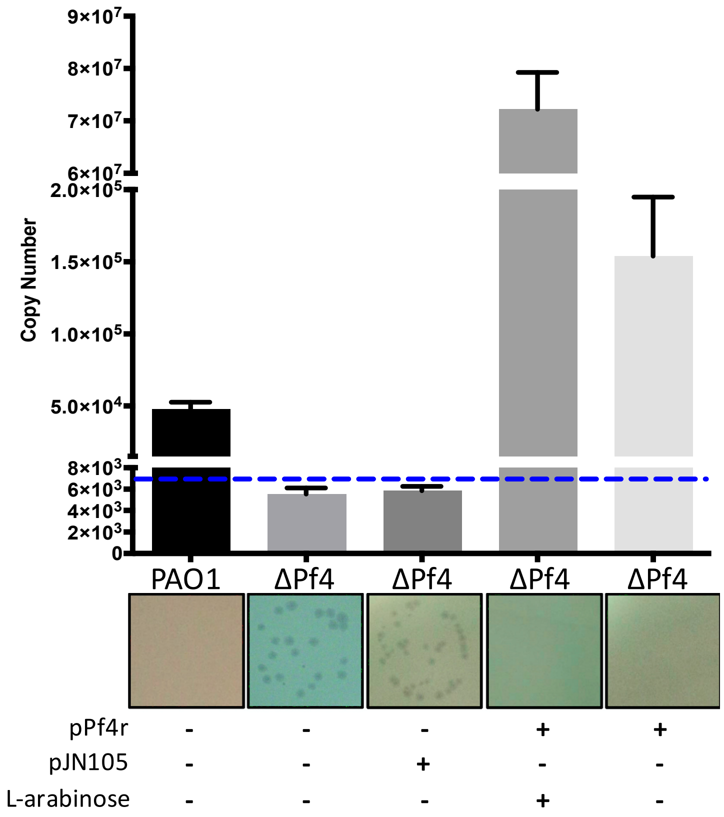

Figure S3. Leaky expression of *pf4r* with the araBAD promoter system
Expression of *pf4r* was determined by qRT-PCR; the blue dashed line represents the detection threshold. Total RNA was extracted from 1 x 10^6 cells in the mid-log phase, converted to cDNA and then normalised to 10 ng/µL. In the qPCR, 1 µL of the normalised cDNA was used as template. Error bars represent standard deviations. Representative images of the observed plaques (if any) are shown below the bars. For all figures, N = 3 and n = 3.

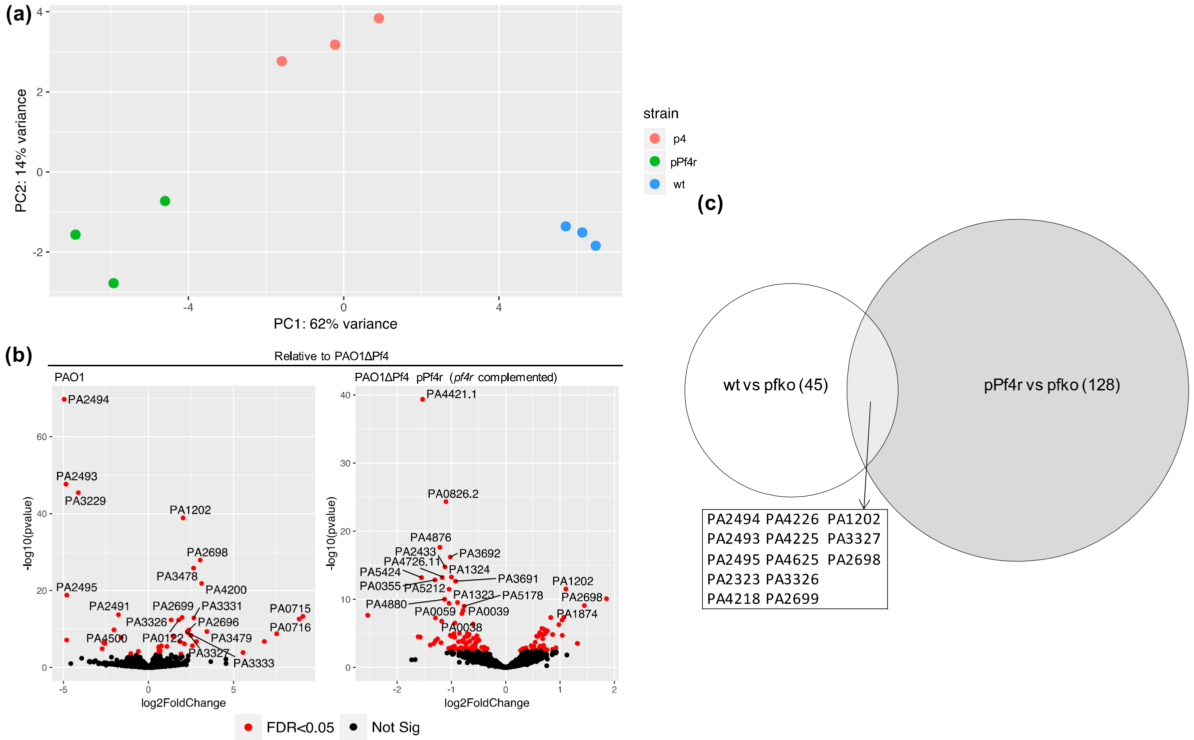

Figure S4. Ordination of complemented strains against the wild type and Pf4 knockout, and comparison of top 20 differentially expressed gene.

a) The samples wild type *P. aeruginosa* (wt, blue), *P. aeruginosa* PAO1ΔPf4 (p4, red) and PAO1ΔPf4 pPf4r (rC, green) were ordinated based on their first two principal components to visualise the overall effect of experimental covariates and batch effects. Each condition was tested using biological triplicates. b) The genes that had a false discovery rate (or adjusted *p* value) < 0.05 relative to PAO1ΔPf4 were labelled red. Genes that were downregulated and upregulated will scatter to the left and right, respectively. The top 20 most differentially expressed genes are indicated in the plot. c) The differentially expressed genes were plot into Euler diagrams using the Eulerr package (version 4.1.0) to help to identify common genes that were differentially expressed.
